# Mutation analysis of *FANCD2*, *BRIP1/BACH1*, *LMO4 *and *SFN *in familial breast cancer

**DOI:** 10.1186/bcr1336

**Published:** 2005-10-21

**Authors:** Aaron G Lewis, James Flanagan, Anna Marsh, Gulietta M Pupo, Graham Mann, Amanda B Spurdle, Geoffrey J Lindeman, Jane E Visvader, Melissa A Brown, Georgia Chenevix-Trench, the Kathleen Cuningham Foundation Consortium for Research into Familial Breast Cancer

**Affiliations:** 1 Department of Cancer Genetics, Queensland Institute of Medical Research, Brisbane, Queensland, Australia; 2 Westmead Institute for Cancer Research, University of Sydney at Westmead Millennium Institute, Westmead Hospital, Westmead, New South Wales, Australia; 3 Peter MacCallum Cancer Centre, Melbourne, Victoria, Australia; 4 VBCRC Laboratory, The Walter and Eliza Hall Institute of Medical Research, Melbourne, Victoria, Australia; 5 School of Molecular and Microbial Sciences, University of Queensland, St Lucia, Queensland, Australia; 6 Department of Pathology, University of Queensland, St Lucia, Queensland, Australia

## Abstract

**Introduction:**

Mutations in known predisposition genes account for only about a third of all multiple-case breast cancer families. We hypothesized that germline mutations in *FANCD2*, *BRIP1/BACH1*, *LMO4 *and *SFN *may account for some of the unexplained multiple-case breast cancer families.

**Methods:**

The families used in this study were ascertained through the Kathleen Cuningham Foundation Consortium for Research into Familial Breast Cancer (kConFab). Denaturing high performance liquid chromatography (DHPLC) analysis of the coding regions of these four genes was conducted in the youngest affected cases of 30 to 267 non-*BRCA1/2 *breast cancer families. In addition, a further 399 index cases were also screened for mutations in two functionally significant regions of the *FANCD2 *gene and 253 index cases were screened for two previously reported mutations in *BACH1 *(p. P47A and p. M299I).

**Results:**

DHPLC analysis of *FANCD2 *identified six silent exonic variants, and a large number of intronic variants, which tagged two common haplotypes. One protein truncating variant was found in *BRIP1/BACH1*, as well as four missense variants, a silent change and a variant in the 3' untranslated region. No missense or splice site mutations were found in *LMO4 *or *SFN*. Analysis of the missense, silent and frameshift variants of *FANCD2 *and *BACH1 *in relatives of the index cases, and in a panel of controls, found no evidence suggestive of pathogenicity.

**Conclusion:**

There is no evidence that highly penetrant exonic or splice site mutations in *FANCD2*, *BRIP1/BACH1*, *LMO4 *or *SFN *contribute to familial breast cancer. Large scale association studies will be necessary to determine whether any of the polymorphisms or haplotypes identified in these genes contributes to breast cancer risk.

## Introduction

Pathogenic mutations in *BRCA1*, *BRCA2*, *TP53*, *PTEN*, *ATM *and *CHEK2 *account for approximately a third of high-risk breast cancer families, suggesting that other breast cancer susceptibility genes exist [1-5]. Given the number of candidate breast cancer susceptibility genes, any approach to their identification needs to be focussed. Genes whose products are known to interact with BRCA1 and/or BRCA2, or are down-regulated in breast tumours, are particularly attractive candidates, and can be prioritised for investigation.

*FANCD2 *is one of eight genes known to cause the fatal human autosomal recessive disorder Fanconi anaemia (FA) [6,7]. FA is a heterogenous condition characterised by progressive bone marrow failure, congenital abnormalities, hypersensitivity to DNA damaging agents and, most importantly, an increased risk of developing cancer [8]. There are currently eight cloned FA genes (*FANCA*, *FANCC*, *FANCD1/BRCA2*, *FANCD2*, *FANCE*, *FANCF*, *FANCG *and *FANCL*), all of which interact with each other in a common cellular pathway [6,7]. Five of the FA proteins (FANCA, C, E, F, and G) form a constitutive complex in the nucleus of normal cells [9]. With the help of the recently identified ubiquitin ligase protein PHF9 (or FANCL), this multisubunit nuclear complex mediates the monoubiquitination of the FANCD2 protein at lysine 561 in response to the S-phase of the cell cycle or DNA damage [6]. The activated FANCD2 protein is then translocated to chromatin and DNA-repair foci, where it co-localises with other DNA repair proteins such as BRCA1, BRCA2, ATM, NBS1 and RAD51 [9]. Interestingly, this translocation has been recently identified to be BRCA1 dependent, suggesting that FANCD2 and BRCA1 interact in this process [6]. In response to ionising radiation, FANCD2 is also phosphorylated by ATM on serine 222, which leads to the activation of an S-phase checkpoint of the cell cycle [9]. *FANCD2 *is located at 3p25.3 and consists of 44 exons, encoding a protein of 1,451 amino acids. Houghtaling *et al*. [10] showed that *FANCD2 *homozygous and heterozygous mice display a high incidence of epithelial tumours, including mammary and ovarian carcinomas. These mice display other features found in *BRCA2 *mutant mice, including germ-cell defects, small size, and perinatal lethality [11]. FANCD2, like BRCA2, may, therefore, play an important role in the recombination DNA repair pathways [10]. The FA pathway has also been implicated in ovarian cancer, as the FANC-BRCA pathway was shown to be disrupted in a subset of ovarian tumour lines [12]. Furthermore, the 3p25-26 region of the human genome has been shown to have a high incidence of loss of heterozygosity in ovarian tumours [13]. Analysis of the FA genes (*FANCA*, *B*, *C*, *D1*, *D2*, *E*, *F*, *G*) in 88 non-*BRCA1*, non-*BRCA2 *breast cancer families failed to identify any penetrant mutations, but none of these families were known to share a haplotype around the relevant *FANC *genes, or to include cases of ovarian cancer [14].

BRIP1/BACH1 was first isolated and identified by using a glutathione S-transferase fusion protein containing the BRCT motifs and the carboxyl terminus of BRCA1. This protein was originally named BACH1 (for BRCA1-associated carboxy-terminal helicase 1), but is also known as BRIP1 (for BRCA1 interacting protein 1) [15]. The *BRIP1*/*BACH1 *gene maps to 17q22 and contains 20 exons, encoding a protein of 1,249 amino acids. Amino acid residues 888 to 1,063 of BRIP1/BACH1 interact with the BRCT domain of BRCA1 during the process of DNA repair [15]. Cantor *et al*. [15] screened the *BRIP1*/*BACH1 *gene for mutations in 21 sporadic breast/ovarian cancer cell lines, and 65 individuals with early onset breast cancer. Two germline heterozygous missense variants (p. P47A and M299I) were detected in the germlines of two early onset breast cancer patients but no family members were available for segregation analysis. Both variants are within the helicase domain of BACH1 (residues 1 to 888), with P47A located in the highly conserved nucleotide binding box, and M299I situated between two other conserved motifs [15]. Two other studies looking at variants in the *BRIP1*/*BACH1 *gene in breast cancer families failed to find any highly penetrant mutations, although these studies were limited in their sample size, and the number of available samples from additional family members, and none of the families were known to share a haplotype around *BRIP1*/*BACH1 *[16,17].

LMO4 is a member of the LIM-only (LMO) family of transcription regulators. The four known members of this group (LMO1 to LMO4) are composed of two LIM domains and are thought to function as transcriptional cofactors via protein-protein interactions (reviewed in [18]). LMO1 and LMO2 overexpression is linked to T-cell tumourigenesis and LMO4 has been associated with breast oncogenesis, where overexpression is observed in approximately 50% of breast cancer cell lines and primary breast cancers [19]. Furthermore, overexpression of LMO4 induces mammary hyperplasia in transgenic mice and may be a predictor of poor outcome in breast cancer [20]. The presence of LMO4 in a complex containing the binding partners Ldb1, CtIP and the familial breast cancer tumour suppressor BRCA1 provides further compelling evidence for LMO4 playing a significant role in breast cancer pathogenesis [21], and activating mutations might be predicted to occur in some tumours and even in the germline of some patients. Although no activation mutations have been found, one somatic truncation mutation of *LMO4 *has been reported in a sporadic breast tumour [22]. This finding, as well as the deregulation of LMO4 expression in breast cancer and the interaction between LMO4 and the tumour suppressor BRCA1, prompted us to screen non-*BRCA1/2 *familial breast cancer cases for genetic alterations in *LMO4 *that may contribute to pathogenesis.

Stratifin (SFN; 14-3-3 σ; HME1) was first identified by serial analysis of gene expression (SAGE) analysis as an epithelial specific marker that was expressed at seven-fold lower levels in breast cancer cells compared to normal breast epithelium [23]. Recently, hypermethylation of *SFN *was detected in more than 90% of invasive breast cancers and was specifically associated with lack of expression [24]. In addition, methylation of this gene was detected in 83% of ductal carcinoma *in situ *and 38% of atypical hyperplasias but was unmethylated in all hyperplasias without atypia and normal breast epithelium obtained from patients without breast cancer [25]. Of most interest was the fact that *SFN *hypermethylation was also detected in the histologically normal adjacent breast epithelium in patients with breast cancer, suggesting that methylation of this gene may be an early event in breast cancer development. *SFN *is a negative regulator of cell cycle progression and is suggested to have an important function in preventing breast tumour cell growth, particularly at the G2 cell cycle checkpoint [26]. BRCA1 is a co-activator of SFN, and the expression of SFN is modulated by the BRCA1 status of the cell and requires intact BRCA1 and p53 to synergistically induce the optimal level of stratifin required for DNA damage response [27]. Interestingly, there is a nine-fold decreased expression of SFN in *BRCA1*- and *BRCA2*-related tumours compared to sporadic breast tumours [28]. SFN is located on 1p36.11 and is encoded by a single 747 base pair (bp) exon; 1p36 is a target of loss of heterozygosity in 16% to 37% of sporadic breast tumours [29,30] and in 32% to 35% of familial tumours [31]. To our knowledge there has been no report of mutation analysis of *SFN *in familial breast cancer.

We sought to carry out mutation analysis of *FANCD2*, *BRIP1*/*BACH1*, *LMO4 *and *SFN *in a large number of non-*BRCA1/*2 breast cancer families. For the biggest genes, *FANCD2 *and *BRIP1*/*BACH1*, we screened a smaller number of families, but included those in which the affected family members shared a haplotype around the gene of interest. We also screened additional index cases for mutations in the *FANCD2 *exons that contain the ATM phosphorylation (S222) and the FANCD2 monoubiquitination regions (K561), and the *BRIP1*/*BACH1 *exons that contained the previously reported breast cancer-association variants, p. P47A and p. M299I.

## Materials and methods

### Multiple-case breast cancer families

Multiple-case breast cancer families were ascertained through the Kathleen Cuningham Foundation Consortium for Research into Familial Breast Cancer (kConFab) [32]. The ascertainment criteria for families without mutations in *BRCA1 *or *BRCA2 *were four or more cases of breast or ovarian cancer (Criteria 1), or two or more if one has 'high risk' features, such as breast cancer diagnosis at less than 40 years, male breast cancer, bilateral breast cancer, or ovarian and breast cancer in the same woman (Criteria 1B). In both cases, the criteria also require that two or more affected women are alive and that the families have four or more living, female, unaffected first or second degree relatives over the age of 18. The index cases, defined as the youngest available breast cancer case, were tested by diagnostic laboratories for mutations in *BRCA1 *and *BRCA2 *by a variety of methods estimated to be 75% sensitive, and a subset were fully sequenced for *BRCA1 *and *BRCA2*.

A subset of the index cases screened for mutations were included in a 10 cM genome-wide search for novel breast cancer susceptibility genes in multiple case breast cancer families from which *BRCA1 *and *BRCA2 *mutations had been excluded by high-sensitivity methods and in which no haplotype was shared at either locus (data not shown). The index cases qualified for *FANCD2 *and *BRIP*/*BACH1 *mutation analysis if an individual family logarithm of the odds (LOD) score under heterogeneity or a non-parametric LOD score of ≥0.5 had been obtained at any of the markers closest to or flanking the *FANCD2 *(D3S1304, D3S1263, D3S2338) or *BRIP*/*BACH *(D17S944, D17S949, D17S787) genes.

All 44 coding exons of *FANCD2 *were evaluated in 33 index cases from 30 non-*BRCA1/2 *multiple case breast cancer families. Three families contained two cases with the same age of onset of breast cancer and so both cases were screened. The families were selected because they contained one or more cases of ovarian cancer (n = 18), or because all of the affected individuals in the family shared a haplotype around the 3p25 region (n = 12). The entire *BRIP1*/*BACH1 *coding sequence (19 exons) was evaluated in the index case of 75 breast cancer families in which all the affected individuals shared a haplotype around *BRIP1*/*BACH1 *on chromosome 17q (n = 7), or which had undergone complete sequencing of *BRCA1 *and *BRCA2 *(n = 68). All three coding exons of *LMO4 *were screened in the index cases from 247 non-*BRCA1/2 *breast cancer families, and the single coding exon of *SFN *was screened in the index cases from 92 non-*BRCA1/2 *breast cancer families. Index cases from an additional 164 families were screened for just 639 bp of the single *SFN *exon. Eight index cases were fully screened for *FANCD2*, *BRIP1*/*BACH1 *and *LMO4 *genes (and six of these for SFN as well), and 227 individuals from 222 families were screened for both *LMO4 *and *SFN*.

In addition, 399 index cases, from 356 non-*BRCA1/2 *breast cancer families (some had more than one index case because multiple women were affected at the same age), were screened for *FANCD2 *mutations in the ATM phosphorylation (exon 9) and the FANCD2 monoubiquitination (exon 19) regions. Of these additional index cases (from 231 families) that were used for additional *FANCD2 *screening, 253 were also screened for *BRIP1*/*BACH1 *mutations in exons 3 and 7, where the p. P47A and p. M299I breast cancer-associated variants are located.

We used as controls DNA from 93 unrelated, adult, female monozygotic twins (only one from each pair) selected from a sample of 3,348 twin pairs. The twins were almost exclusively of European origin and had been recruited through the Australian Twin Registry. Approvals were obtained from the Human Research Ethics Committees of the Queensland Institute of Medical Research, and for kConFab from the Peter MacCallum Cancer Centre and all other committees to which kConFab reports.

### Mutation analysis

Primers were designed using the web-based program Primer3 [33] to amplify 43 amplicons covering the 44 exons of *FANCD2 *[GenBank: NT005927], 21 amplicons covering 19 exons of *BRIP1*/*BACH1 *gene [GenBank: NT010783.13], three amplicons for the three exons of *LMO4 *[GenBank: NM 006769] and three amplicons for the single coding exon of *SFN *[GenBank: NM006142]. PCR products were amplified from 15 ng of genomic DNA using AmpliTaq Gold (PE Applied Biosystems, Forest City, CA, USA) in a final volume of 20 μL. The amplification of fragments was optimised as needed by adjusting the MgCl_2 _concentration, adding 1 M Betaine, or by lowering the annealing temperature.

For *LMO4*, cycling conditions for the exon 2 and exon 4 amplicons were 94°C for 12 minutes, followed by four sets of four cycles of 94°C for 30 s, 63°C to 57°C for 45 s and 72°C for 30 s, with the annealing temperature dropping 2°C after each set of four cycles, followed by 30 cycles of 94°C for 30 s, 55°C for 45 s and 72°C for 30 s, and a final extension of 72°C for 7 minutes. Cycling conditions for exon 3 were 94°C for 12 minutes, followed by four sets of 4 cycles of 94°C for 30 s, 72°C to 66°C for 45 s and 72°C for 30 s, with the annealing temperature dropping 2°C after each set of 4 cycles, followed by 30 cycles of 94°C for 30 s, 64°C for 45 s and 72°C for 30 s, and a final extension of 72°C for 7 minutes. The same four-step touchdown protocol used for the amplification of *LMO4 *was also used for screening *FANCD2*, *BACH1 *and *SFN *(Table 1). The *SFN *exon was screened in three PCR fragments. SFN1a and SFN1b.1 were screened by denaturing high performance liquid chromatography (DHPLC) in 267 index cases. SFN1b.2 could not be screened successfully using DHPLC and so a subset of 92 cases, chosen based on DNA availability, were sequenced directly for this amplicon.

Amplicons were then denatured at 95°C for five minutes and cooled to 60°C over 30 minutes (1°C/minute) prior to injection onto the Varian Helix System (Varian, Walnut Creek, CA, USA). DHPLC was carried out at the recommended melt temperature for each exon (Table 1) as determined by the Stanford melt algorithm [34,35]. Analysis of the DHPLC results was performed using the Star Workstation version 5 (Varian). Samples that produced a heterozygous peak or an aberrant shift in retention time and/or peak shape were confirmed by DHPLC and re-amplified for sequencing. DNA sequencing was performed with both forward and reverse primers using the ABI Prism Big Dye Terminator cycle Sequencing Ready reaction kit (PE Applied Biosystems) and analysed on an ABI 377 sequencer. Coding variants and variants located near the exon/intron boundary, were analysed *in silico *for amino acid changes, conservation in the mouse homologue (UCSC Genome Bioinfomatics [36]), predicted splicing defects (BDGP Splice Site Prediction [37], SpliceSiteFinder [41], and ESE Finder [38]), and predicted mRNA folding changes (mFOLD [39]).

All available family members' DNA samples were genotyped for any missense and frameshift variants, and for variants that appeared to lose a splice site, or have exonic splicing enhancer and/or mRNA folding changes, as predicted by the above web-based programs. Frameshift variants, missense variants, or variants predicted to affect splicing were further screened by DHPLC in 93 controls. Individuals carrying the rare *FANCD2 *variants, c. 633 C>T, c. 1828+34 C>T, c. 2148 C>G, c. 2021+10 G>T, and c. 3558 C>G, were also sequenced for the common c. 694+17 G>C variant in exon 9 to determine on which haplotype these rare variants occurred.

## Results

### *FANCD2*

DHPLC analysis of *FANCD2 *in the 33 index cases from 30 breast and ovarian cancer families, and of exons 9 and 19 (containing the ATM phosphorylation site and the FANCD2 monoubiquitination site, respectively) in a further 399 non-*BRCA1/2 *index cases, identified 32 germline sequence alterations, most of which were novel (Table 2). Analysis of sequencing results identified 25 intronic variants, 6 silent coding variants, and another variant located within the 3' untranslated region (UTR).

The c.633 C>T and c. 2148 C>G variants did not appear by *in silico *analyses to affect mRNA folding or the concensus splice site sequences, as predicted by the BDGP Splice Site Prediction, SpliceSiteFinder, and mFOLD web-based programs (Table 3). c.2148 C>G was predicted to change the SF2/ASF exon enhancer sites and gain a SRp55 enhancer site. Because this nucleotide is not conserved in the murine *Fancd2 *gene, however, the functional significance of these changes remains unclear. The c. 3558 C>G (L1186L) variant, located 3 bp 5' of the end of exon 35, was predicted to result in a gain of a SC35 exonic splicing enhancer site, and a loss of a SF2/ASF site, and also subtly changing the predicted mRNA folding. In addition, the BDGP Splicing program predicted that the variant causes a complete loss of the donor site for exon 35 splicing, although this was not predicted by SpliceSiteFinder, consistent with the more sensitive algorithm of the BDGP splicing program [37]. To address this further we performed RT-PCR analysis with lymphoblastoid cell line RNA but found no evidence for altered splicing of this transcript (data not shown). The c. 3558 C>G variant was found in a family with five cases of breast cancer, of whom two also had ovarian cancer. DNA was available from two additional affected relatives of the index case (her daughter and cousin). The variant was carried by the daughter (affected at age 27 years) but not by the cousin (affected at age 34 years), nor by any of the four unaffected female relatives (ages 22 to 57 years), nor by four male unaffected relatives. The c. 3558 C>G variant was not found using DHPLC in any of 93 matched twin controls. The other *FANCD2 *coding variants, c. 1122 A>G, c. 1440 T>C, c. 1509 C>T, and the 3' UTR variant c. 4359 C>T were all common and/or previously reported as a single nucleotide polymorphism (SNP) and, therefore, no *in silico *analyses were conducted.

Two common haplotypes of *FANCD2 *were identified, represented by c. 379-6 delTT, c. 694+17 C>G, c. 784-19 C>T, c. 990-38 C>G, c. 1122 A>G, c. 2270-28 G>T, c. 2976+36 T>C, c. 3850-203 C>T, c. 4185+33 T>C, and c. 4281+97 A>G (Table 4). We were unable to calculate the exact frequencies of each of the haplotypes because DHPLC did not distinguish the two homozygotes from each other. Sequencing showed that the rare variants, c. 633 C>T, c. 1828+34 C>T, c. 2148 C>G, c. 2021+10 G>T and c. 3558 C>G, were all found on the common haplotype that corresponds to the reference sequence found on the NCBI database [42].

### *BRIP1/BACH1*

A total of 10 nucleotide variants, four of which have not been previously reported, were identified in *BRIP1*/*BACH1 *among 75 non-*BRCA1/2 *index cases (Table 5). Six of these variants were exonic, of which one was a single base-pair deletion, four resulted in amino acid substitutions and one was silent (Table 5). Three of the missense variants, c. 430 G>A (p. A144T), c. 584 T>C (p. L195P) and c. 3464 G>A (p. G1155E), and the deletion variant c. 3401delC were absent in 93 controls. The c. 584 T>C (p. L195P) variant has been reported previously in an early onset breast cancer case, but not in controls [17]. *In silico *analyses of c. 430 G>A (p. A144T), c. 584 T>C (p. L195P) and c. 3464 G>A (p. G1155E) predicted that they may affect mRNA folding and exonic splicing enhancers, but have no affect on the consensus splice sites (Table 3). The 3' UTR variant c. 3782T>C was predicted to cause the addition of a stem loop at the position of the variant. This nucleotide is not conserved in the murine *Bach1*/*Brip1 *gene, however, so may not, therefore, be functionally significant. The 3401delC variant was predicted to cause a frameshift resulting in a premature stop codon 15 amino acids downstream. This would truncate the protein by 100 amino acid residues. The intronic changes and the other exonic variants, c. 517 C>T (p. R172C) and c. 3411 T>C (p. T1137T), were all found in at least one control, indicating that they are likely to be benign polymorphisms.

DNA samples from additional family members of the c. 430 G>A, c. 584 T>C, c. 3464 G>A, and the c. 3401delC carriers were available for analysis, but none of these variants were found to segregate with breast cancer in the families. Both the c.430 G>A and c.3401delC variants were inherited from the father of the index case who had no personal or family history of breast cancer, and not from the affected mother with a strong family history. The c.584 T>C variant was identified in only the unaffected father and uncle of the index case, and not in 24 other relatives (including two other affected females). Finally, the c.3464 G>A variant was found in a family in which the index case shared a haplotype around *BRIP1*/*BACH1 *with four affected maternal relatives, but only the index case carried the variant, indicating that it was inherited from her father who had no personal or family history of breast cancer.

Analysis of exons 3 and 7 of *BRIP1*/*BACH1 *in a further 253 non-*BRCA1*/*BRCA2 *breast cancer index cases did not identify the p. P47A and p. M299I variants previously reported, or any other variants.

### *LMO4*

Index cases from 247 families were screened by DHPLC across the three coding exons of *LMO4*. Using the primers designed to amplify exon 3, two intronic variations were observed in two individuals each, c.237-72T>G and c.237-51_237-46delTTCTTT, but no coding variants were identified.

### *SFN*

DHPLC analysis of most (639/747 bp) of the coding exon of *SFN *of the youngest available member affected with breast cancer of each of 256 families identified one silent variant, c.621 C>T (T207T) in 23 index cases. One individual was found to carry three missense alterations (c.594C>A (F198L), c.653C>A (L218I) and c.730C>A (Q244K)). In the 92 individuals screened for the remaining 89 bp *SFN *coding region, 10 different alterations were observed in the 3' UTR in 11 cases (c.748G>C, c.765C>T, c.766C>A, c.767C>T, c.775C>A, c.776C>T, c.777C>T, c.786C>A, c.787C>A, and c.792C>T). Several of these were predicted to affect the secondary structure of the corresponding transcript (Table 3).

## Discussion

Previous analyses of *FANCD2 *and *BRIP1*/*BACH1 *in non-*BRCA1/2 *families failed to identify any pathogenic mutations [14,16,17]; however, these studies did not choose the families to be screened on the basis of haplotype sharing, or the occurrence of other cancers (e.g. ovarian cancer in the case of *FANCD2*) in the family. Furthermore, some of these studies were limited by the fact that DNA from additional family members was not available for genotyping. To our knowledge, *LMO4 *and *SFN *have not been previously examined as *BRCAx *candidate genes. We therefore hypothesized that germline mutations in the *BRCA1*-interacting genes, *FANCD2*, *BRIP1*/*BACH1*, *LMO4 *and *SFN*, may account for some non-*BRCA1/2 *multiple-case breast cancer families. *LMO4 *and *SFN *are both small genes and so 247 and 267 index cases, respectively, were screened for these two genes. Because of the large size of the *FANCD2 *and *BRIP1*/*BACH1 *genes, however, we screened a smaller number of non-*BRCA1*/2 breast cancer families for mutations in these genes (30 and 75 families, respectively), but they were selected on the basis of all the available affected individuals sharing a haplotype around *FANCD2 *or *BRIP1*/*BACH1*, having at least one case of ovarian cancer (*FANCD2*), or having had full sequence analysis of *BRCA1 *and *BRCA2 *(*BRIP1*/*BACH1*). In addition, we screened a further 399 and 253 cases, respectively, for specific regions of the *FANCD2 *and *BRIP1*/*BACH1 *genes that contained functionally important domains, or variants previously found in the germline of breast cancer cases.

DHPLC analysis the *FANCD2 *gene indicated a high level of conservation in the coding sequence, indicated by a paucity of missense changes. Only six coding variants (c. 633 C>T, 1122 A>G, 1440 T>C, 1509 C>T, 2148 C>G and 3558C>G) were identified in the *FANCD2 *gene, all of which were silent. c.633 C>T and 2148 C>G did not appear to dramatically affect the predicted RNA splicing or folding by *in silico *analyses and were, therefore, assumed to be neutral SNPs. 3558 C>G (L1186L) was suggested by the BDGP Splice site predictor program (but not SpliceSiteFinder) to result in the complete loss of the donor site, possibly resulting in the misplicing of exon 35. RT-PCR analysis of a lymphoblastoid cell line from the carrier failed, however, to identify any aberrant transcripts, suggesting that this variant is unlikely to be pathogenic. The L1186L variant was not identified in any of 93 controls, but it did not segregate with breast cancer in the single family in which it was found. Therefore, this variant was also assumed to be a rare, neutral SNP.

Two common haplotypes of the *FANCD2 *gene were identified, one of which (haplotype B) was identical to the reference sequence obtained from the NCBI database. All of the rare variants were found to occur on haplotype B. Even though individually these variants were classified as neutral SNPs, an association study designed to test whether the two haplotypes confer different breast cancer risks would be worthwhile.

In the analysis of the *BRIP1*/*BACH1 *gene, we did not observe the two previously reported variants, p. P47A and p. M299I, in the 253 non-*BRCA1/2 *breast cancer cases [15]. However, we did identify three non-conservative missense variants (p. A144T, p. L195P, and p. G1155E) and one novel frameshift mutation (c. 3401delC) in the 75 selected non-*BRCA1*/*BRCA2 *breast cancer index cases. None of these variants were found in 93 controls. Additional genotyping of a total of 68 family members indicated, however, that these variants are not the underlying cause of breast cancer in these families, as none of the other affected relatives carried the variants. Nevertheless, it is possible that these variants are low-risk breast cancer susceptibility alleles, in which case further investigation may be warranted. The 3782 T>C 3' UTR variant of *BRIP1*/*BACH1 *is predicted to alter the folding of the transcript; however, the biological significance and frequency of this change in the normal population has yet to be determined.

Mutation analysis of 82 sporadic tumours previously revealed one somatic frameshift mutation of *LMO4 *[22]. No activating or inactivating coding or splice site mutations of *LMO4 *were found by DHPLC analysis of 247 index cases from non-*BRCA1/2 *families. Two intronic variants were found, each in two index cases. Their recurrent nature in two families and apparent lack of effect on splicing suggests that they are rare SNPs.

SFN is markedly down-regulated in breast cancer tissue compared to normal mammary epithelium but to our knowledge has not been evaluated for germline mutations in familial breast cancer. We screened the majority of the coding region of the gene in 267 index cases and found one silent change, T207T, in 23 index cases. This silent change has been previously reported (rs11542704) [43]. We also found some variants in the 3' UTR, and three different missense changes in one individual (F198L, L218I and Q244K) that were considered unlikely to be pathogenic because of the multiple occurrences in one individual. None of these variants have been previously reported.

## Conclusion

Mutation analysis of the *BRCA1*-interacting genes *FANCD2*, *BRIP1*/*BACH*, *LMO4 *and *SFN *in a large number of non-*BRCA1/*2 breast cancer families did not identify any highly penetrant, pathogenic mutations. Given that DHPLC is a robust and sensitive screening technique, we consider it unlikely that we missed any coding or splice site pathogenic mutations among the index cases analysed. In particular, we analysed each PCR fragment at all the temperatures recommended by the DHPLC melt algorithm and under these conditions DHPLC has been reported to have a sensitivity of 99.4% [40]. It appears unlikely, therefore, that *FANCD2*, *BRIP1*/*BACH*, *LMO4 *and *SFN *account for more than a small proportion of inherited forms of breast cancer. Many novel SNPs were identified in these genes, however, and large association studies of breast cancer cases and controls is warranted to determine whether any of these variants confer small risks of breast cancer.

## Abbreviations

bp = base pair; DHPLC = denaturing high performance liquid chromatography; FA = Fanconi anaemia; kConFab = Kathleen Cuningham Foundation Consortium for Research into Familial Breast Cancer; RT-PCR = reverse-transcription polymerase chain reaction; SFN = stratifin; SNP = single nucleotide polymorphism; UTR = untranslated region.

## Competing interests

The authors declare that they have no competing interests.

## Authors' contributions

GCT, GL, JV and JF were responsible for the design of this study and GCT, JF and AL drafted the manuscript. AL, JF, and AM performed the experimental work. Haplotype sharing data were provided by GP and GM. Clinical and genetic data, and biospecimens, were provided by kConFab. MAB assisted with analysis of mFOLD data, and with preparation of the manuscript.

## Figures and Tables

**Table 1:** *FANCD2*, *BRIP1/BACH1*, *LMO4 *and *SFN *primers

Gene	Exon	Forward primer	Reverse primer	Annealing temp (°C)	Amplicon size (bp)	DHPLC temp (°C)
*FANCD2*	1	GGTGAGCCCAGCTTATTGC	GCCTCGAGAGACTACGACCA	55	188	64
	2	CTTCAGCAACAGCGAAGTAGTCTG	GATTCTCAGCACTTGAAAAGCAGG	50	421	56
	3	GGACACATCAGTTTTCCTCTC	GAAAACCCATGATTCAGTCC	50	308	56
	4 & 5	TCATCAGGCAAGAAACTTGG	GAAGTTGGCAAAACAGACTG	50	466	53, 58
	6	GAGCCATCTGCTCATTTCTG	CCCGCTATTTAGACTTGAGC	50	282	56
	7	CAAAGTGTTTATTCCAGGAGC	CATCAGGGTACTTTGAACATTC	50	342	55, 60
	8	GACCAGAAAGGCTCAGTTCC	CTGCAGCACATTACCCAGAA	55	463	51, 56
	9	CAGCTCTGCATTTCACACGTA	TGCAGTGATAGAACCCCCATA	55	265	60, 62
	10	TGCCCAGCTCTGTTCAAACC	AGGCAATGACTGACTGACAC	50	219	59
	11	TGCCCGTCTATTTTTGATGAAGC	TCTCAGTTAGTCTGGGGACAG	50	391	56
	12	TCATGGTAGAGAGACTGGACTGTGC	ACCCTGGAGCAAATGACAACC	55	432	53, 58
	13	ATGGCAGGAACTCCGATCTT	CCTTATTCAGCATGCCCTGT	55	363	56
	14	GGGCATGCTGAATAAGGTGT	GACAGTGGGAAGGCAAGCTA	55	189	57
	15	GGGAGTGTGTGGAACAAATGAGC	AGTTTCTACAGGCTGGTCCTATTCC	50	513	56
	16	AACGTGGAATCCCATTGATGC	TTTCTGTGTTCCCTCCTTGC	58	378	57
	17	GATGGTCAAGTTACACTGGC	CACCTCCCACCAATTATAGTATTC	54	381	57
	18	CTATGTGTGTCTCTTTTACAGGG	AATCTTTCCCACCATATTGC	50	233	52, 57
	19	CATACCTTCTTTTGCTGTGC	CCACAGAAGTCAGAATCTCCACG	48	198	59
	20	ACACACCGGGGAATGTTGT	CTGCAGGTTGGGTGACAGAG	53	341	49, 54, 59
	21	GAGTTTGGGAAAGATTGGCAGC	TGTAGTAAAGCAGCTCTCATGC	54	231	59
	22	ACACTCTGCACTGCCCTTTT	GAAAGAAGCTGTGGGACTGG	55	239	60
	23	TGGTTTTCCCTGTAGCCTTG	CTCAACTTCCCCACCAAGAG	55	294	60
	24	TTGTGTTCCCTATGTATGTGGAG	TTGATCTGCCCTTTCCCTAA	54	269	58
	25	CAGCTAGCTCCAGAGGCAAC	TGCACAATAAGTGAAACATCCT	54	229	58
	26	TTCATAGACATCTCTCAGCTCTG	GTTTTGGTATCAGGGAAAGC	52	283	52, 57
	27	GCCATGCTTGGTAATTTTGG	TTCCAATTACTGATGCCATGAT	55	319	57
	28	TTGTTTTCTGAGGGCAATGA	TGGGATGTCACAAACCTTCA	55	230	52, 57
	29	GGTCTTGATGTGTGACTTGTATCCC	CCTCAGTGTCACAGTGTTCTTTGTG	58	446	51, 56
	30	CATGAAATGACTAGGACATTCC	CTACCCAGTGACCCAAACAC	52	280	60
	31	GACTCCATTGCGAACCCTTA	CAGCCAAAAATGAAACCTCA	55	260	58
	32	TGAGGTTTCATTTTTGGCTGA	GTGCCTTGGTGACTGTCAAA	55	268	58
	33	TTGATGGTACAGACTGGAGGC	AAGAAAGTTGCCAATCCTGTTCC	50	273	58
	34	AGCACCTGAAAATAAGGAGG	GCCCAAAGTTTGTAAGTGTGAG	52	342	52, 57
	35	TGTTAGACCGGGAACGTCTT	TTGGCTCATTGGAAGAAACC	55	257	54, 59
	36	TCTTCCAATGAGCCAAATAGC	CCCAGCCTGATGATGATTCT	55	450	58
	37	AGAGGTAGGGAAGGAAGCTAC	CCAAAGTCCACTTCTTGAAG	50	232	60
	38	GATGCACTGGTTGCTACATC	CCAGGACACTTGGTTTCTGC	52	274	58
	39	ACACTCCCAGTTGGAATCAG	CTTGTGGGCAAGAAATTGAG	54	369	54, 59
	40	TGGGCTGGATGAGACTATTC	CCAAGGACATATCTTCTGAGCAAC	52	222	59
	41	TGATTATCAGCATAGGCTGG	GATCCCCCAATAGCAACTGC	52	270	54, 59
	42	CATTCAGATTCACCAGGACAC	CCTTACATGCCATCTGATGC	54	226	59
	43	AACCTTCTCCCCTATTACCC	GGAAAATGAGAGGCTATAATGC	52	434	58
	44	TGTATTCCAGAGGTCACCCAGAGC	CCAGTAAGAAAGGCAAACAGCG	59	234	58
						
*BRIP1/ BACH1*	2	TTCTTTGTAAGGCGTGTCTCAA	ATACTCAATGTACTTTATGGGTCA	50	246	55
	3	TTGTTGTGTTCCCTGGAGTG	GACAGCATGGCTGAACCAGT	55	325	56
	4	CTGGGTGAACTGGGCTGTAG	CAACTGACCCAGGCAAAATA	55	281	52, 57
	5	AAGCCTTACATCCTGGTTCC	CTAACTGGGTTATTTACTGCCAAT	54	254	55
	6	GAGCTGTTTTGGCCTTTGAG	TTCCTTCTTTAAAACTGAACAATGG	55	239	56
	7	TTCCATGTGAGGTTTGATAACG	GCAGTTAATTTGATTTTCCGAAG	55	481	53, 58
	8	GCCTTGTGGCTTTAATGATG	CATCTCCATGAGTAGGAAGAAGGT	55	373	57
	9	TGAAATATCTTGCCTGCTGTTG	TTTTTCATATAAAGGCAGCACAA	55	321	52, 57
	10	GATCAACGCATGACAATAATGA	AATTCACTAAATACGTTTCACAGGT	50	270	55
	11	CCCTCCCAACCCCTCTATAC	TGCTAGCATCCAAATTAGGCTAT	55	258	54
	12	TGGGAAGGTACCAGCTCTTT	TGCTGGTACTGAGCAAGAAGAC	55	333	56
	13	TCAGACTCCTAGACTCAAGCGAT	TTACTTGCTGGCACTTCAGG	54	364	54, 59
	14	CATGCCTTTTTCAGGCATAA	TTGCCTCTACCCTAGGAAGC	50	318	53, 58
	15	GCCGTAGTCACATTGGCTTA	TTTTCACCAGACCATGAAATAA	50	278	56
	16	TTCTGAGAATTTTGGATATGCTTTT	TGCTGAAAGACTTGCACAATG	55	319	57
	17	TTGAGATCACACAGCTGATTAGTTA	CCAGTTCCTATGGTTCCAGTT	55	458	50, 55
	18	CTGTCCCACTGGAAAACTGG	TCTGCTGTGAAATACTGTGCTT	50	297	54
	19	GGTTACTTCACTAGAAAAAGCAAGTG	TCAAAGGTAAATGGGAAGAACTTT	55	456	56
	20a	GCAATTATGTTAGCTAGGAGCAGA	TCAAAATCTCTATTTGAAGTGGACTG	55	516	52, 57
	20b	TGAACATCCGCTCTGTTCTG	TGCAATCCTCAGCTTTCACT	55	305	55
	20c	AACAATTCAGATTGCATTTTAGC	GAGTTTAACATAAGCATGATGAC	50	328	53
						
*LMO4*	2	TTTCTCTCCCTGTCCCCTTC	CCCCATCTCCAGGAAAGAC	55	304	59,64
	3	CCAAAGGGATGCCCAGAGT	GCCCCCATGTAGGTAGGAAA	64	254	55,60
	4	CCTTGTTTTCAGTGGGTTTG	GGTTGAACCTTCCAACTTGC	55	246	59
						
*SFN*	1a	GCCAAGAGCAGGAGAGACAC	ATGCGCTTCTTGTCGTCAC	60	510	63
	1b.1	GAGAGCCGGGTCTTCTACCT	TGATGAGGGTGCTGTCTTTG	62	316	62
	1b.2	TCTCTCTGGCCAAGACCACT	TCCCACCCCATACTAGTCCTC	64	249	63

DHPLC, denaturing high performance liquid chromatography.

**Table 2:** Mutation Analysis of *FANCD2*

Segment	Nucleotide change^a^	Protein effect	No. of heterozygous cases	Frequency in controls (n = 93)	Reported in SNPper^b ^or FA database
-	-82 G>A		1/33	Not tested	No
-	-155 G>A		1/33	Not tested	No
-	-157 G>A		9/33	Not tested	No
-	-158 C>T		1/33	Not tested	No
-	-160 G>A		1/33	Not tested	No
Intron 5	379-6 del TT		11/33	Not tested	No
Exon 9	633 C>T	I211I	1/432	Not tested	No
Intron 9	694+17 G>C		127/432	Not tested	No
Intron 10	784-19 C>T		11/33	Not tested	No
Intron 12	990-38 C>G		11/33	Not tested	No
Exon 14	1122 A>G	V374V	11/33	Not tested	Yes
Intron 16	1414-9 C>T		N/A	Not tested	No
Intron 16	1414-23 T>C		N/A	Not tested	No
Intron 16	1414-89 T>A		N/A	Not tested	Yes
Intron 16	1414-117 A>T		N/A	Not tested	No
Intron 16	1414-125 G>A		N/A	Not tested	No
Intron 16	1414-136 C>T		N/A	Not tested	No
Exon 17	1440 T>C	H480H	N/A	Not tested	Yes
Exon 17	1509 C>T	N503N	N/A	Not tested	Yes
Intron 21	1828+34 C>T		2/33	Not tested	No
Intron 22	2021+10 G>T		1/33	Not tested	No
Intron 22	2021+31 C>T		16/33	Not tested	Yes
Intron 22	2021+100 A>G		1/33	Not tested	No
Exon 23	2148 C>G	T716T	1/33	Not tested	No
Intron 24	2270-28 G>T		11/33	Not tested	No
Intron 30	2976+36 T>C		11/33	Not tested	Yes
Intron 30	2977-39 C>T		1/33	Not tested	No
Exon 35	3558 C>G	L1186L	1/33	0/93	No
Intron 38	3850-203 C>T		11/33	Not tested	No
Intron 42	4185+33 T>C		11/33	Not tested	Yes
Intron 43	4281+97 A>G		11/33	Not tested	No
3' UTR	4359 C>T	3' UTR	15/33	Not tested	No

^a^GenBank accession number NT005927. ^b^Variants have been previously reported in the SNP database SNPper [44] or Fanconi Anemia (FA) Mutation Database [45]. UTR, untranslated region.

**Table 3:** *In silico* analysis of novel synonymous and 3' UTR alterations in *FANCD2*, *BRIP1/BACH1 *and *STN*

Gene	Sequence variant	Location	Conserved in mouse^a^	Predicted effect on mRNA structure^b^	Predicted effect on consensus splice sites^c^	Predicted effect on exonic splice site^d^
*FANCD2*	633C>T	Exon 9	Yes	None	None	No change
	2148C>G	Exon 23	No	None	None	Gains SRp55, changes SF2/ASF
	3558C>G	Exon 35	No	Slight shortening of side branch	Loss of exon 35 donor^e^	Gains SC35, loses SF2/ASF
*BRIP1/ BACH1*	430 G>A	Exon 5	Yes	None	None	Loses SRp55 and SRp40, gains SF2/ASF
	584T>C	Exon 6	No	Completely changes mRNA structure	None	Loses SRp40
	3401 delC	Exon 20	Yes	None	None	No change
	3464 G>A	Exon 20	No	Loss of one cluster of stem loops, gain of another	None	Loses SRp40 and SF2/ASF
	3782T>C	3' UTR	No	Additional stem loop in middle of structure		
*SFN*	748G>C	3' UTR	No	Change in branch position		
	765C>T	3' UTR	Yes	Change in branch position		
	766C>A	3' UTR	Yes	Loss of major side branch		
	767C>T	3' UTR	Yes	No change		
	775C>A	3' UTR	Yes	No change		
	776C>T	3' UTR	Yes	No change		
	777C>T	3' UTR	Yes	No change		
	786C>A	3' UTR	Yes	Change in branch position		
	787C>A	3' UTR	Yes	No change		
	792C>T	3' UTR	Yes	No change		

^a^According to UCSC Genome Bioinfomatics [36]. ^b^According to mFOLD [39].

^c^According to SpliceSiteFinder [41] and BDGP Splice Site Prediction [37].

^d^According to ESE Finder [38]. ^e^According to BDGP Splice Site Prediction [37], but not SpliceSiteFinder [41]. UTR, untranslated region.

**Table 4:** *FANCD2 *haplotypes

Haplotype		Common or rare
A	- - CTGGTCTCGCCGCC	Common
B	TTGCCAGTCTACCGCC	Common
C	TTGCCAGTCTATCGCC	Rare
D	TTGCCAGTCTACTGCC	Rare
E	TTGCCAGTCTACCTCC	Rare
F	TTGCCAGTCTACCGGC	Rare
G	TTGCCAGTCTACCGCG	Rare

The two common haplotypes were distinguishable by the single nucleotide polymorphisms c. 379-6 delTT, c. 694+17 C>G, c. 784-19 C>T, c. 990-38 C>G, c. 1122 A>G, c. 2270-28 G>T, c. 2976+36 T>C, c. 3850-203 C>T, c. 4185+33 T>C, and c. 4281+97 A>G. The rare haplotypes all occurred on the haplotype B background and were represented by the rare variants, c. 633 C>T, c. 1828+34 C>T, c. 2021+10 G>T, c. 2148 C>G, and c. 3558 C>G. Letters A-G represent the seven different *FANCD2 *haplotypes.

**Table 5:** Mutation analysis of *BRIP1/BACH1*

Segment	Nucleotide change^a^	Protein effect	No. of heterozygous cases	Controls	Reported in SNP per^b^
Intron 4	379-28 G>A		1/75	1/93	Yes
Exon 5	430 G>A	A144T	1/75	0/93	No
Intron 5	508-31C>G		23/75	26/93	Yes
Exon 6	517C>T	R173C	1/75	1/93	Yes
Exon 6	584T>C	L195P	1/75	0/93	Yes
Intron 12	1795-47 C>G		27/75	Not tested	Yes
Exon 20	3401 delC	frameshift	1/75	0/93	No
Exon 20	3411 T>C	T1137T	40/75	34/93	Yes
Exon 20	3464 G>A	G1155E	1/75	0/93	No
Exon 20	3782 T>C	3' UTR	1/75	Not tested	No

^a^GenBank accession number NT010783.13. ^b^Variants have been previously reported in the SNP database SNPper [42]. UTR, untranslated region.
